# Differential Control of Interleukin-6 mRNA Levels by Cellular Distribution of YB-1

**DOI:** 10.1371/journal.pone.0112754

**Published:** 2014-11-14

**Authors:** Sujin Kang, Taeyun A. Lee, Eun A. Ra, Eunhye Lee, Hyun jin Choi, Sungwook Lee, Boyoun Park

**Affiliations:** Department of Systems biology, College of Life Science and Biotechnology, Yonsei University, Seoul, South Korea; German Cancer Research Center, Germany

## Abstract

Cytokine production is essential for innate and adaptive immunity against microbial invaders and must be tightly controlled. Cytokine messenger RNA (mRNA) is in constant flux between the nucleus and the cytoplasm and in transcription, splicing, or decay; such processes must be tightly controlled. Here, we report a novel function of Y-box-binding protein 1 (YB-1) in modulating interleukin-6 (IL-6) mRNA levels in a cell type-specific manner. In lipopolysaccharide (LPS)-stimulated macrophages, YB-1 interacts with IL-6 mRNA and actively transports it to the extracellular space by YB-1-enriched vesicles, resulting in the proper maintenance of intracellular IL-6 mRNA levels. YB-1 secretion occurs in a cell type-specific manner. Whereas macrophages actively secret YB-1, dendritic cells maintain it predominantly in the cytoplasm even in response to LPS. Intracellular YB-1 has the distinct function of regulating IL-6 mRNA stability in dendritic cells. Moreover, because LPS differentially regulates the expression of histone deacetylase 6 (HDAC6) in macrophages and dendritic cells, this stimulus might control YB-1 acetylation differentially in both cell types. Taken together, these results suggest a unique feature of YB-1 in controlling intracellular IL-6 mRNA levels in a cell type-specific manner, thereby leading to functions that are dependent on the extracellular and intracellular distribution of YB-1.

## Introduction

The immune response comprises a variety of processes in response to infection or tissue damage; immune cells and soluble mediators, such as cytokines of the innate and adaptive immune system, play important roles in the host defense mechanism. The inflammatory response is generally a protective reaction and maintains tissue homeostasis [1]. Although an uncontrolled response causes chronic inflammation, scarring, and fibrosis, inflammation in a normal context results in a complete resolution of the response and return to the local homeostatic state after pathogen elimination or tissue repair [2].

YB-1 is a member of a large family of cold shock domain-containing proteins and is involved in inflammatory processes [3], [4]. YB-1 consists of the alanine/proline-rich N-terminal domain (A/P domain), the cold shock domain (CSD), and the large C-terminal domain (CTD) with alternating clusters of positively and negatively charged amino acids. The consensus sequences of the CSD mediate YB-1 interactions with DNA and RNA, and the CTD of YB-1 is associated with the majority of its protein partners [5]. The N-terminal A/P domain binds to actin microfilaments, which contribute to mRNA localization [3]. YB-1 has been identified as a pleiotropic protein that participates in DNA repair, pre-mRNA splicing, the regulation of transcription and translation, and mRNA packing and stability [6]. It can be secreted from the cell to perform extracellular functions, acting as an extracellular mitogen [7]. The multiple activities of YB-1 are exemplified by its involvement in cell proliferation and differentiation, the stress response, and inflammatory responses [4].

The importance of the balance between stabilization and degradation of cytokine mRNA is illustrated by the differences between inflammatory diseases and immune homeostasis [8], [9]. Cytokine mRNA decay is tightly regulated at the post-transcriptional level through *cis*- or *trans*-acting elements [10], where cytokine transcripts are transiently stabilized and then undergo regulated degradation. However, the precise mechanisms controlling cytokine mRNA metabolism remain to be elucidated.

In this study, we demonstrate a distinct role of YB-1 in the tight regulation of intracellular IL-6 mRNA levels in a cell type-specific manner. YB-1 is secreted from macrophages but not dendritic cells after inflammatory stimuli and interacts with IL-6 mRNA. YB-1-depleted macrophages exhibit increased intracellular IL-6 mRNA levels, whereas dendritic cells exhibit decreased IL-6 mRNA expression after YB-1 depletion. In macrophages, the amount of intracellular IL-6 mRNA is controlled by YB-1 secretion, which enables cytosolic IL-6 mRNA to be exported into the extracellular fluid. In contrast, intracellular YB-1 enhances IL-6 mRNA stability in dendritic cells. These findings illustrate the distinct function of YB-1 as a critical regulator in controlling intracellular IL-6 mRNA levels differentially in a cell type-specific manner.

## Results

### YB-1 is secreted in a cell type-dependent manner

**Figure 1:**
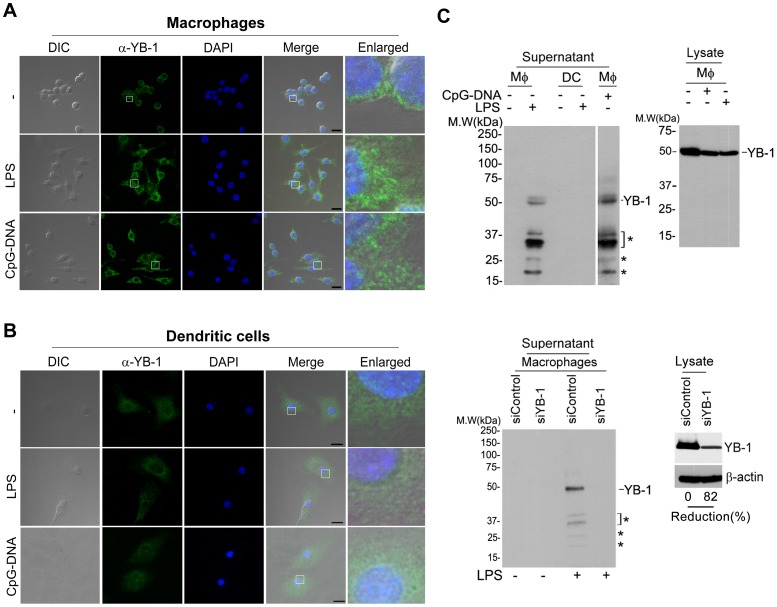
Inflammatory stimuli induce YB-1 secretion in a cell type-specific manner. (**A**) Immunofluorescence microscopy assay (IFA) of YB-1 in RAW macrophages following exposure to LPS (80 ng/ml) or CpG-DNA (1 µM). LPS or CPG-DNA-stimulated macrophages exhibited YB-1-enriched exporting vesicles. 6.5× digital enlargement of main image, Scale bars, 10 µm. (**B**) Bone marrow-derived Dendritic cells (BMDC) were stimulated with LPS (80 ng/ml) or CpG-DNA (1 µM). Anti-YB-1 antibody with Alexa 488-conjugated secondary antibody and DAPI were used. YB-1-enriched vesicles were not detected in BMDC. 6.5× digital enlargement of main image, Scale bars, 10 µm. (**C**) Western blot analyses of YB-1 in macrophages and dendritic cells with anti-YB-1antibody. TCA-precipitated extracellular supernatant from macrophages contained secreted YB-1, which was confirmed by depletion of YB-1. Lineage markers were used to characterize macrophages or dendritic cells (Fig. S1). Asterisk indicates the molecular weight species of extracellular YB-1. Data are representative of three (**A–B**), or two (**C**) experiments.

Studies have shown that YB-1 exhibits various subcellular localization patterns depending on the stimulus [7], [11]. In particular, human monocytes stimulated with LPS secrete YB-1 from micro-vesicles [7]. We began examining the role(s) of YB-1 in the immune response by reproducing documented observations of YB-1 secretion in response to LPS, which promotes robust cytokine production to induce innate and adaptive immunity [7]. First, we assessed YB-1 subcellular localization in LPS-stimulated macrophages and dendritic cells. As shown previously, we observed intracellular secretory vesicle formation with enriched levels of endogenous YB-1 in LPS-treated macrophages, but not in unstimulated cells (**Figure 1A**). To explore TLR-agonist specificity in YB-1 secretion, we measured the pattern of intracellular vesicles in macrophages exposed to CpG-DNA, which activates the TLR9 signaling cascade. In CpG-DNA-stimulated macrophages, YB-1 secretory vesicles were clearly detected to an extent similar to LPS (**Figure 1A**). Interestingly, unlike macrophages, YB-1 was distributed throughout the cytoplasm, with one or two speckles close to the nuclear membrane in LPS- or CpG-DNA-stimulated bone marrow-derived dendritic cells (BMDCs) (**Figure 1B**). Therefore, YB-1 exhibits differential subcellular distribution that varies according to cell type, resulting in the regulation of distinct biological functions in macrophages and dendritic cells.

To confirm these findings, we examined YB-1 secretion from macrophages and dendritic cells following LPS stimulation. Equal numbers of macrophages and dendritic cells were exposed to LPS or CpG-DNA for 24 h and then the supernatants were immunoblotted with anti-YB-1 antibody. Macrophages showed secretion of YB-1, whereas no release of cytosolic protein was observed from dendritic cells (**Figure 1C** **, upper left panels**). The actual molecular weight of YB-1 is 35.9 kDa; however, this intracellular protein exhibits an electrophoretic mobility of ∼50 kDa. Interestingly, the molecular mass of intracellular YB-1 was detectable at 50 kDa but the majority of extracellular YB-1 exhibited the ∼37 kDa size along with several other molecular weight species, indicating that YB-1 may undergo fragmentation processing at different sites in LPS-stimulated macrophages (**Figure 1C** **, asterisks**). Because YB-1 knockdown resulted in no detectable protein bands in the immunoblot assay, it was confirmed that the protein bands observed were not due to non-specific proteins (**Figure 1C** **, bottom panel, asterisk**). Therefore, we conclude that YB-1 is secreted in a cell type-specific manner.

### The localization of YB-1 affects IL-6 production

**Figure 2:**
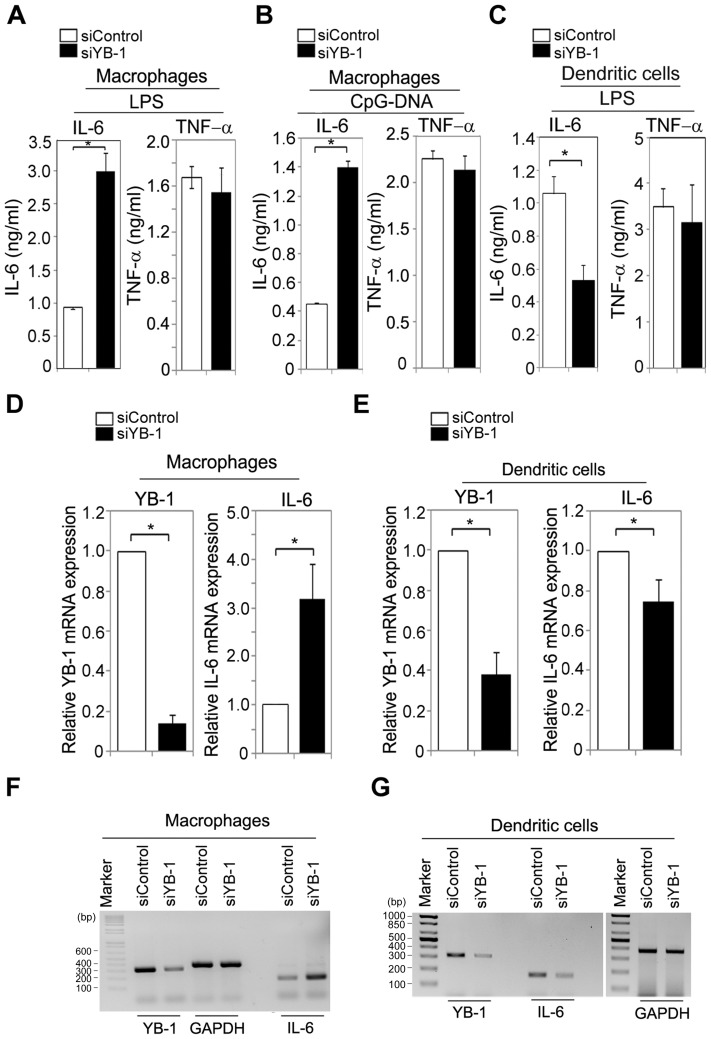
Depletion of YB-1 affects IL-6 mRNA production in a cell type-specific manner. (**A–C**) The increased production of IL-6 was observed in YB-1-depleted macrophages but not BMDC. After depletion of YB-1, macrophages were stimulated with LPS (80 ng/ml) or CpG-DNA (1 µM). ELISA was performed to measure IL-6 and TNF-α levels in RAW macrophages (**A** and **B**) and BMDC (**C**). *P<0.001 (student's t-test) (**D–G**) YB-1 knockdown led to increased IL-6 mRNA levels in macrophages, but reduced in BMDC. IL-6 mRNA levels were assessed by qRT-PCR (**D–E**, primer pairs: P2, P3, and P5 in **Table S1**) or RT-PCR (**F–G**, primer pairs: P1, P3, and P5) in YB-1-depleted macrophages (**D** and **F**) or BMDC (**E** and **G**), stimulated with LPS (80 ng/ml). *P<0.001 (student's t-test). Data are representative of at least three independent experiments.

Because YB-1 serves as a regulator in both pro- and anti-inflammatory responses [4], [12] and its cellular distribution was quite different between macrophages and dendritic cells, we investigated whether this differential localization of YB-1 affects its ability to induce cytokine production in response to LPS. YB-1 was depleted in macrophages or BMDCs using YB-1-specific RNA interference (RNAi). Unexpectedly, depletion of YB-1 enhanced LPS-induced production of IL-6 but not TNF-α in macrophages (**Figure 2A**). Likewise, CpG-DNA-stimulated macrophages expressing YB-1-specific RNAi exhibited increased production of IL-6 but not TNF-α (**Figure 2B**). In contrast to macrophages, LPS-exposed dendritic cells expressing YB-1 RNAi reduced IL-6 production but not TNF-α (**Figure 2C**). To confirm these findings, we examined the effect of YB-1 depletion on IL-6 mRNA production by quantitative RT-PCR (qRT-PCR) in macrophages and dendritic cells. The quantity of IL-6 mRNA produced upon LPS stimulation was increased in YB-1-depleted macrophages (**Figure 2D**) and was decreased in dendritic cells under the same conditions (**Figure 2E**). Similarly, RT-PCR confirmed that YB-1 affected IL-6 mRNA production in macrophages and dendritic cells in opposite ways (**Figure 2F and 2G**). Therefore, we conclude that YB-1-mediated IL-6 mRNA production is dependent upon cell type and tightly co-regulated with differential localization patterns of YB-1.

### YB-1 negatively regulates intracellular IL-6 mRNA levels in macrophages

**Figure 3:**
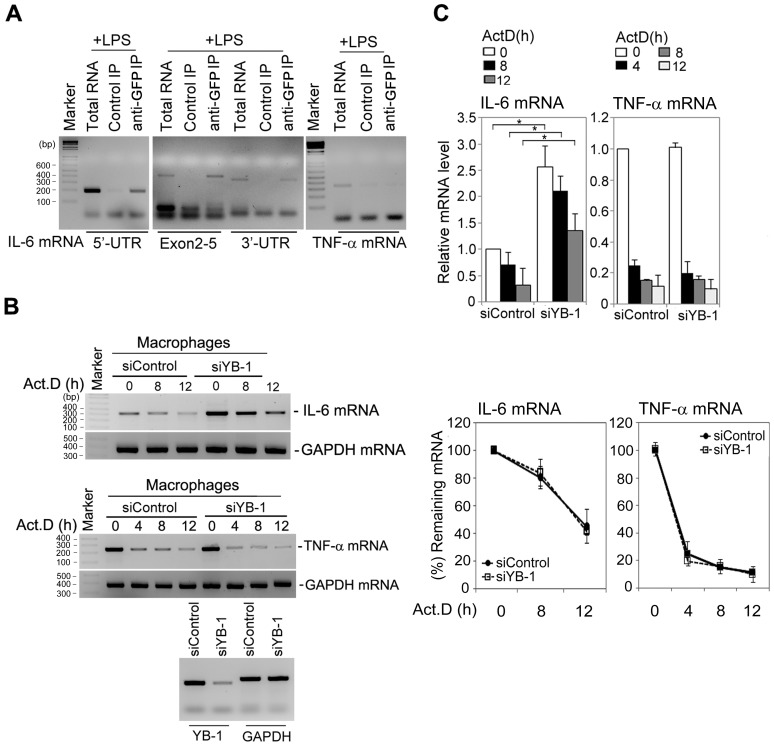
YB-1 interacts with IL-6 mRNA but does not affect IL-6 mRNA stability. (**A**) Macrophages were exposed to LPS (80 ng/ml) and then subjected to RIP assay using appropriate primer pairs (P4, P6, P8, and P9 in **Table S1**). YB-1 bound IL-6 mRNA, but not TNF-α mRNA. (**B**) The effect of YB-1 depletion on IL-6 or TNF-α mRNA stability in LPS-stimulated macrophages. Total mRNA was isolated at different time points after actinomycin D (6 µg/ml) treatment and each RT-PCR was analyzed by the indicated primer pairs (P4, P5, and P8). (**C**) Each graph represents densitometric analysis of RT-PCR data. Relative densitometric values for each mRNA were corrected by dividing each value by that for the GAPDH mRNA in each blot. *P<0.001 (student's t-test). Data are representative of at least three independent experiments.

YB-1 binds near the cap structure of mRNA and also interacts with granulocyte monocyte colony stimulating factor (GM-CSF) mRNA [13], [14]. YB-1 consists of three major domains: a glycine-rich N-terminal domain, a C-terminal domain containing alternating charged amino acids, and a highly conserved cold shock domain [4], [5], [15]. The glycine-rich and cold shock domains of YB-1 are thought to bind nucleic acid, based on previous reports and our results. Therefore, we determined whether YB-1 could bind IL-6 mRNA by conducting IL-6 mRNA pull-down analysis with the indicated primers using LPS-stimulated macrophages. We demonstrated that YB-1 interacts with IL-6 mRNA but not TNF-α mRNA (**Figure 3A**). Because YB-1 depletion increased IL-6 production, the ability of YB-1 to bind IL-6 mRNA suggested a possible role of YB-1 in regulating IL-6 mRNA stability. To investigate this possibility, we treated LPS-stimulated macrophages with the transcriptional inhibitor actinomycin D (Act.D) and then examined IL-6 mRNA stability by RT-PCR. We found that the amount of IL-6 mRNA during ActD exposure was much higher than in control macrophages, but the half-life of IL-6 mRNA in YB-1-depleted macrophages was similar to that of control macrophages. YB-1 depletion did not affect TNF-α mRNA expression or stability (**Figure 3B**). Calculation of mRNA band intensities also indicated that in YB-1-depleted macrophages, the relative amount of IL-6 mRNA increased more than two fold compared to control cells (**Figure 3C** **, upper graphs**); however, we found that the half-life of IL-6 mRNA or TNF-α mRNA was unaffected (**Figure 3C** **, bottom graphs**). These results suggest that YB-1 negatively regulates intracellular IL-6 mRNA expression levels, but it is not due to an increase in RNA turnover.

### YB-1 actively exports cytosolic IL-6 mRNA to the extracellular space to control intracellular IL-6 mRNA levels in macrophages

**Figure 4:**
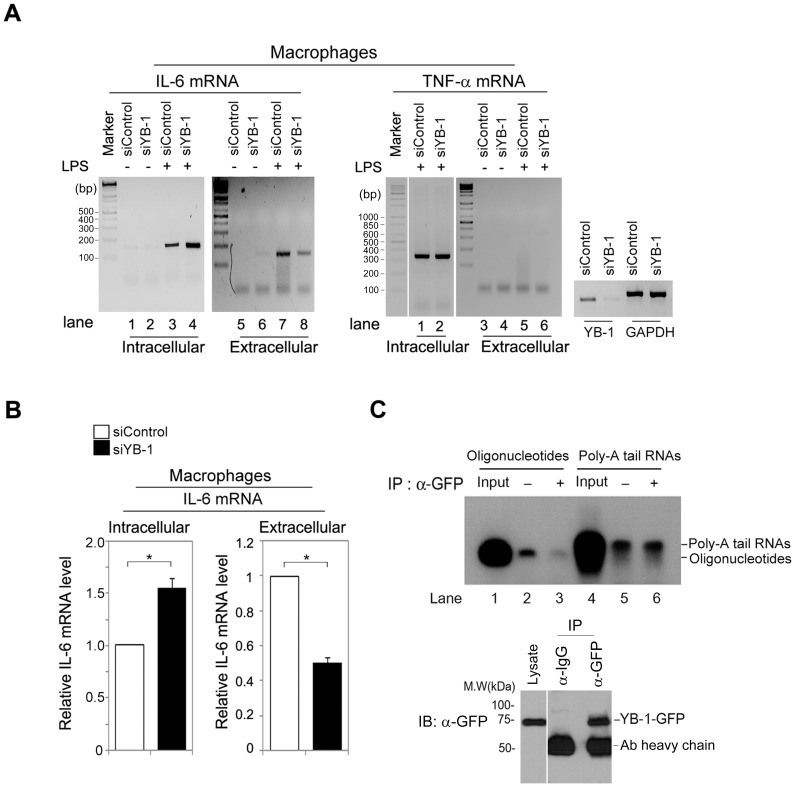
YB-1 is essential for maintaining intracellular IL-6 mRNAs levels by secreting mRNA to the extracellular space in macrophages. (**A**) After LPS stimulation for 24 h, total RNAs were purified separately from medium or cell lysates and the presence of IL-6 or TNF-α mRNAs was examined by RT-PCR. (**B**) The ratio of IL-6 mRNA between intracellular and extracellular fluid was correlated to YB-1 expression level. *P<0.005 (student's t-test). (**C**) YB-1 does not act as an exoribonuclease enzyme. Cell lysates from macrophages stably expressing YB-1-GFP were immunoprecipitated with anti-GFP antibody. YB-1-GFP proteins were then purified and incubated with the P^32^-labeled single-stranded 21mer oligonucleotides or 12mer Poly-A tail RNAs. Exonuclease or exoribonuclease assay was performed at 37°C for 2 h and terminated by adding 2× sample buffer and reaction products were separated on a 15% polyacrylamide 7 M urea gel. YB-1-GFP proteins were probed by anti-GFP-antibody. Data are representative of three (**A, B**), or two (**C**) experiments.

Previous studies have shown that YB-1 associates with GM-CSF mRNA and thereby protects it from degradation, resulting in the development of allergic asthma by accumulating eosinophils in the lung parenchyma and airways [13], [16]. However, our results showed that YB-1 depletion clearly increases IL-6 mRNA levels by a mechanism other than enhancing its stability in macrophages (**Figure 2**). Therefore, we hypothesized that in macrophages, YB-1 may facilitate IL-6 mRNA export to the extracellular space by YB-1 secretory micro-vesicles, resulting in a reduction of total cytosolic IL-6 mRNA levels. It is known that extracellular RNA (exRNA) species present outside the cells from which were transcribed, but their biological function is not fully understood [17], [18]. To explore this possibility, we determined whether IL-6 mRNA exists extracellularly and whether or not secretory YB-1 functions in exporting IL-6 mRNA from cytosol to the extracellular space. Macrophages expressing either control or YB-1 RNAi were incubated in serum-free medium in the absence or presence of LPS for 24 h. Culture medium was collected from the macrophages and then assayed for IL-6 mRNA by RT-PCR. Surprisingly, we found that IL-6 mRNA was secreted to the extracellular space from macrophages (**Figure 4A** **, lane 7 of left panel**); in contrast, we did not observe any secreted TNF-α mRNA (**Figure 4A** **, lane 5 of right panel**). Furthermore, intracellular and extracellular IL-6 mRNA levels correlated with YB-1 expression levels in macrophages. YB-1 depletion increased the amount of intracellular IL-6 mRNA and decreased levels of extracellular IL-6 mRNA (**Figure 4A** **, comparing lane 3 with 4 and lane 7 with 8 of left panel**). TNF-α mRNA levels were unaffected, as shown previously (**Figure 4A, right panel**). We quantified the IL-6 mRNA band intensities from RT-PCR and showed that IL-6 mRNA increased in the intracellular space by 51% in YB-1-depleted macrophages and extracellular IL-6 mRNA levels decreased by 58% (**Figure 4B**).

Because YB-1 binds to single-stranded cisplatin-modified Y box DNA sequences and degrades DNA by its exonuclease activity [19], we investigated whether YB-1 could also degrade RNA. We purified YB-1 by immunoprecipitation and then incubated the protein with oligonucleotides or poly-A tail RNAs. An *in vitro* degradation assay showed that YB-1 degraded oligonucleotides but not RNA (**Figure 4C** **, comparing lane 2 with 3 and lane 5 with 6**), indicating that YB-1 does not have an exoribonuclease activity. Taken together, we demonstrate that YB-1 tightly controls intracellular IL-6 mRNA levels by binding and exporting cytosolic IL-6 mRNA to the extracellular fluid, but does not act as an exoribonuclease.

### Intracellular YB-1 is essential for IL-6 mRNA stability in dendritic cells

**Figure 5:**
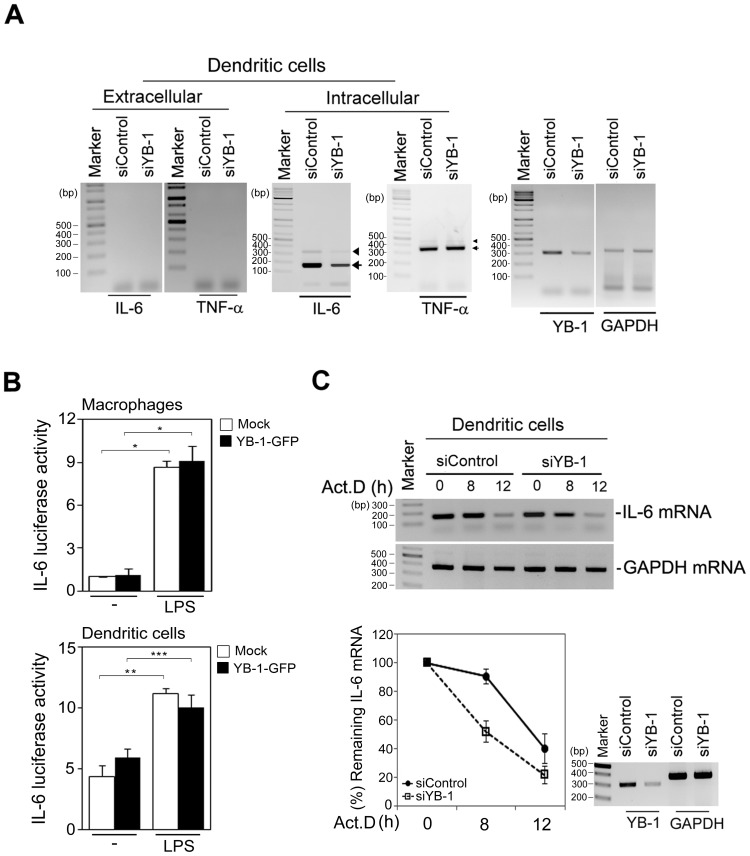
YB-1 has a distinct role in dendritic cells, capable of enhancing IL-6 RNA stability. (**A**) Total RNAs were purified from culture medium or cell lysates from LPS-stimulated BMDCs expressing either control or YB-1 RNAi. The extracellular or intracellular IL-6 mRNA or TNF-α mRNA was examined by RT-PCR (primer pairs: P4 and P7 in **Table S1**). YB-1 knockdown and RNA quantitation were determined by RT-PCR using the indicated primers (primer pairs: P3 and P5). Arrowheads and arrows indicate pre-mRNA and mRNA, respectively. (**B**) Macrophages or dendritic cells were cotransfected with empty vector or YB-1-GFP together with IL-6 and a *Renilla* luciferase reporter gene and then stimulated with LPS for 12 h. Cell lysates were prepared and then measured by *Renilla*-Firefly luciferase dual assay. Luciferase activity was determined using a luminometer. All luciferase assays were performed in triplicate for each reporter construct. (**C**) The effect of YB-1 depletion on IL-6 mRNA stability in LPS-stimulated dendritic cells was assessed by RT-PCR analysis of IL-6 mRNA levels (primer pairs: P1 and P5). Total mRNA was isolated at different time points after ActD treatment. Each graph represents densitometric analysis of RT-PCR data. Relative densitometric values for each mRNA were corrected by dividing each value by that for the GAPDH mRNA in each blot. *P<0.0005, ** *P*<0.001, *** *P*<0.005 (Student's t-test). Data are representative of three independent experiments.

Because YB-1 is involved in regulating transcription, RNA processing, and mRNA stabilization [14], [20], [21], we next investigated whether intracellular YB-1 controls IL-6 mRNA metabolism in dendritic cells. To examine the effects of YB-1 on IL-6 mRNA transcription and splicing, dendritic cells expressing YB-1 RNAi were stimulated with LPS and then IL-6 mRNA production was measured. YB-1 depletion in dendritic cells resulted in reduced IL-6 mRNA production following LPS stimulation. In contrast, TNF-α mRNA levels were unaffected. In addition, IL-6 pre-mRNA accumulation was not observed, suggesting that YB-1 is not involved in IL-6 pre-mRNA processing (**Figure 5A**). To explore whether YB-1 activates IL-6 gene expression, we examined luciferase activity in YB-1-expressing macrophages or dendritic cells using an *IL-6* promoter luciferase reporter. The luciferase assay showed no significant effect by YB-1 on *IL-6* promoter activity (**Figure 5B**), indicating that YB-1 may not be involved in regulating IL-6 mRNA transcription but rather its stability. Indeed, we found that the half-life of IL-6 mRNA in YB-1-depleted dendritic cells was less than in control dendritic cells (**Figure 5C**). Taken together, we conclude that cytosolic YB-1 controls IL-6 production by regulating IL-6 mRNA stability in dendritic cells.

### LPS induces YB-1 acetylation, which differentially regulates HDAC6 expression between macrophages and dendritic cells

**Figure 6:**
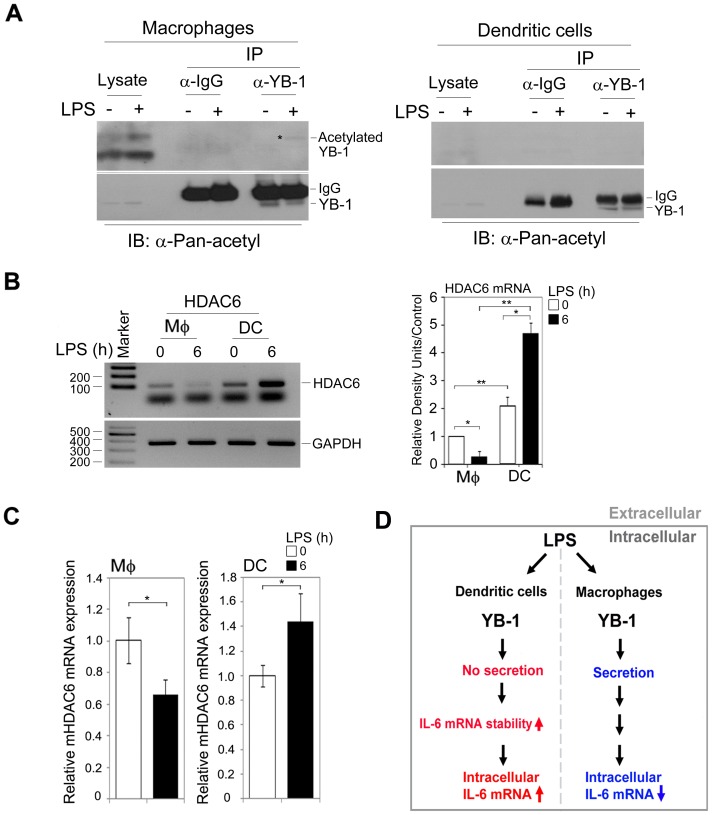
YB-1 acetylation leads to differential regulation of HDAC6 between macrophages and dendritic cells. (**A**) Equal numbers of macrophages and dendritic cells were exposed to LPS for 12 h and then lysed. The lysates were immunoprecipitated with anti-YB-1 antibody and then immunoblotted with anti-Pan-acetyl or anti-YB-1 antibody. (**B and C**) Macrophages or dendritic cells were stimulated with LPS for 0 h or 6 h before total RNA was purified. The expression level of HDAC6 was examined by RT-PCR or qRT-PCR (primer pairs: P10 and P5). *P<0.001, **P<0.005 (Student's t-test). Data are representative of two (**A**) or three (**B, C**) experiments. (**D**) Schematic model of YB-1 controlling intracellular IL-6 mRNA levels in a cell type-specific manner. The proper maintenance of intracellular IL-6 mRNA expression is dependent on the extracellular and intracellular distribution of YB-1.

Based on previous observations that YB-1 acetylation is essential for its secretion [7], we examined this post-translational modification of YB-1 in LPS-stimulated macrophages or dendritic cells. Immunoprecipitation of intracellular YB-1 using anti-YB-1 antibody followed by immunoblotting with anti-Pan-acetyl antibody revealed weak but detectable YB-1 acetylation in macrophages but not in dendritic cells (**Figure 6A**).

Studies have demonstrated that LPS regulates the expression of several members of the HDAC family in macrophages in a time-dependent manner [22]. In particular, the expression of HDAC6 was highly expressed in non-stimulated macrophages, but its expression was significantly reduced after 2 hours of LPS stimulation in macrophages. Therefore, we hypothesized that LPS blocks the activity of HDAC6, allowing intracellular YB-1 to be efficiently acetylated for its secretion. We performed RT-PCR or qRT-PCR assays to examine HDAC6 expression in LPS-stimulated macrophages and dendritic cells over time. Indeed, the levels of HDAC6 decreased in LPS-treated macrophages (**Figure 6B**). Interestingly, in contrast to macrophages, the expression of HDAC6 during LPS stimulation was highly increased in dendritic cells (**Figure 6B**). Similarly, qRT-PCR confirmed that LPS affected HDAC6 mRNA production in macrophages and dendritic cells in opposite ways (**Figure 6C**). These results demonstrate that LPS negatively regulates the expression of HDAC6 in macrophages; in contrast, it positively regulates the expression of both HDACs in dendritic cells, indicating that different expression patterns of HDAC6 may differentially control YB-1 acetylation and secretion in a cell type-dependent manner.

## Discussion

These studies describe a critical role of YB-1 in controlling intracellular IL-6 mRNA levels in a cell type-specific manner. In macrophages responding to inflammatory stimuli, YB-1 is actively secreted, allowing it to bind IL-6 mRNA and promote the export of intracellular IL-6 mRNA to the extracellular fluid, thereby maintaining immune homeostasis by reducing excess IL-6 mRNA within the cell. However, the function of YB-1 in dendritic cells is distinct from that in macrophages. Dendritic cells do not exhibit YB-1 secretion in response to LPS. Instead, cytosolic YB-1 is essential for IL-6 mRNA stability as a positive regulator. Therefore, YB-1 is needed for IL-6 mRNA production to protect against microbial infection. Moreover, YB-1 is involved in maintaining intracellular IL-6 mRNA levels to prevent a hyperactive immune response. These distinct functions are dependent on the subcellular distribution of YB-1 (**Figure 6D**). Depending on the context of cell type-dependent YB-1 function, our data imply that there may be potential relevance to the differences in YB-1 function between the inflammatory infiltrating macrophages and tissue-resident macrophages during various immune responses.

Several reports have documented different post-translational modifications for YB-1, including fragmentation, acetylation, and phosphorylation. Interestingly, we show that secretion of YB-1 is dependent on cell type, and that the molecular mass of intracellular and extracellular YB-1 is quite different. The molecular mass of intracellular YB-1 is mostly detectable at ∼50 kDa, whereas the majority of extracellular YB-1 exhibits a size of approximately 37 kDa, along with several other molecular weight species. Previous reports have shown that YB-1 is cleaved by the 20S proteasome, which allows its truncated form to translocate to the nucleus, resulting in more efficient protection of cells from DNA damage [23]–[25]. In particular, the 18-kDa fragment of secreted YB-1 was detected in the plasma of cancer patients [26] and we also show the 18 kDa fragment in TCA-precipitated extracellular supernatant from LPS-stimulated macrophages. Therefore, it is possible that the various YB-1 fragments that form in response to LPS may result in altered subcellular localization and immunological function.

Furthermore, YB-1 acetylation is required for its secretion [7]. Our results show that YB-1 is acetylated in LPS-stimulated macrophages, but not in dendritic cells. In addition, we demonstrate that HDAC6 expressions differ in a cell type-dependent manner, indicating that differences in YB-1 secretion between macrophages and dendritic cells may be correlated with differential HDAC6 expression. In addition, during the early phase of inflammation, YB-1 is phosphorylated at Ser102, a site located in the highly conserved cold-shock domain [12]. In addition, calcineurin-mediated YB-1 dephosphorylation regulates CCL5 expression during monocyte differentiation [27]. Because YB-1 contains several possible sites for phosphorylation, it may be possible that LPS regulates YB-1 phosphorylation, leading to changes in its subcellular localization.

YB-1 can function as a negative or positive regulator on RNA metabolism [20], [28]. For example, heterozygous YB-1 knockout mice show increased basal expression levels of CXCL1 in the kidney and liver, whereas LPS stimulation results in decreased CXCL1 expression in these organs. Interestingly, the peritoneal lavage fluid of these mice treated with LPS contains elevated CXCL1 levels as compared with wild-type mice. Our study also shows that YB-1 exhibits different functions depending on the cell type, capable of controlling intracellular IL-6 mRNA levels through export or enhancement of stability.

Several types of extracellular RNAs have been described. Recent studies have shown that microRNAs (miRNAs) are released and that secretory miRNAs are transferable by packaged vesicles and functional in recipient cells [29], [30]. It is possible that secreted IL-6 mRNA may be packaged with YB-1-containing exosomal vesicles and transferred to neighboring cells, resulting in IL-6 production in these cells without LPS stimulation. In particular, our results imply that YB-1 could also regulate the secretion or stability of other mRNA species.

Because misregulation of IL-6 mRNA expression levels contributes to autoimmune and chronic inflammatory diseases, an understanding of the regulatory proteins involved in controlling cytokine mRNA levels is essential for the development of new classes of immunomodulatory therapies.

## Materials and Methods

### Cell lines

Murine RAW 264.7 macrophages (ATCC TIB-71) were cultured in DMEM supplemented with 10% heat inactivated fetal bovine serum (HyClone, Logan, UT) and penicillin/streptomycin (Hyclone). Cells were grown at 37°C in humidified air with 5% CO_2_.

### Generation of BMDC

Bone marrow-derived dendritic cells (BMDC) were generated from wild-type C57BL/6 mice (Orient Bio, Gyeonggi-do, South Korea), in medium containing 5 ng/ml GM-CSF (BioLegend, San Diego, CA). Briefly, femurs and tibiae were collected from 4-week-old mice. After removing bone-adjacent muscles, marrow cells were extracted by flushing with a 25-gauge needle. Bone marrow cells were then resuspended in DMEM (10% FBS and 1% antibiotics) with GM-CSF (5 ng/ml). Fresh medium was replenished on Days 2 and 4. BMDC were generated after 6–8 days of culture. Mice were maintained under pathogen-free conditions according to guidelines set by the committee for animal care at the Yonsei University.

### Reagents

LPS (E. coli 026:B6) and 1826-CpG DNA (5′-TsCsCsAsTsgsAsCsgsTsTsCsCsTsgsAsCsgsTsT-3′) were purchased from Sigma (St. Louis, MO) and TIB Molbiol (Berlin, Germany), respectively. YB-1 antibodies were obtained from Cell Signaling Technology (Danvers, MA) and Santa Cruz Biotechnology (Santa Cruz, CA). Actinomycin D (Act. D) and Trichloroacetic acid (TCA) were purchased from Sigma.

### Retroviral transduction and RNAi production

HEK 293T cells were transfected with plasmids encoding VSV-G and Gag-Pol, as well as either shRNA for YB-1 or shRNA for GFP (Control). Thirty-six to forty-eight hours post-transfection, media containing viral particles were collected, filtered through a 0.45 µm membrane and incubated with RAW macrophages for 24 h. Cells were selected with puromycin. The shRNA sequence against YB-1 was annealed and subcloned into the pSUPER retroviral vector (Oligoengine, Seattle, WA) using the following primers: 5′-GATCCGGTCATCGCAACGAAGGTTTTCTCGAGAAAACCTTCGTTGCGATGACCTTTTTTGGAAA-3′ and 5′-AGCTTTTCCAAAAAA GGTCATCGCAACGAAGGTTTTCTCGAGAAAACCTTCGTTGCGATGACC-3′. The siRNA sequence against GFP, which was used as a negative control, was cloned using the following primers: 5′-GATCCGCAAGCTGACCCTGAAGTTCCTCGAGGAACTTCAGGGTCAGCTTGCTTTTTTGGAAA-3′ and 5′-AGCTTTTCCAAAAAAGCAAGCTGACCCTGAAGTTCCTCGAGGAACTTCAGGGTCAGCTTGCGG-3′.

### ELISA and siRNA transfection

Cells were treated with 80 ng/ml LPS or 1 µM CpG-DNA for 12 h. The media were collected and mouse IL-6 and TNF-α levels were analyzed by ELISA according to the manufacturer's recommendations (BD Biosciences, San Jose, CA). BMDC were transfected (DharmaFECT; Thermo scientific, Rockford, IL) with either scrambled siRNA (Control) or YB-1 siRNA for 48 h. The siRNA sequences were as follows: YB-1 (5′- UCAUCGCAACGAAGGUUUUTT-3′, 5′- AAAACCUUCGUUGCGAUGATT-3′) and negative control (5′-UUCUCCGAACGUGUCACGUTT-3′, 5′-ACGUGACACGUUCGGAGAATT-3′).

### Immunofluorescence assay (IFA)

For immunofluorescent staining, cells were fixed in 3.7% formaldehyde and permeabilized with 0.1% Triton X-100 prior to incubation with anti-YB-1 antibody and Alexa Fluor 488-conjugated secondary antibody (Life technologies, Carlsbad, CA). DAPI (4′, 6-diamidino-2-phenylindole) (Sigma) was used as a nuclear counterstain. Cells were imaged using a fluorescence microscope.

### RNA Immunoprecipitation (RIP assay)

Cytosolic fractions were isolated from macrophages expressing YB-1-GFP using a hypotonic buffer containing 10 mM HEPES, pH 7.9, 15 mM MgCl_2_, 10 mM KCl, 0.05% NP-40, protease inhibitor cocktail (Roche, Mannheim, Germany), and 100 units of RNase inhibitor (TaKaRa, Otsu, Japan). After incubation with anti-GFP antibody and protein G-Sepharose beads (Sigma), samples were washed with ice-cold RIP buffer (150 mM KCl, 25 mM Tris-Cl, pH 7.4, and 0.5% NP-40) and RNA was extracted using an RNA purification kit (GeneAll, Seoul, South Korea) according to the manufacturer's instructions.

### Purification of YB-1 protein

Macrophages stably expressing YB-1-GFP were lysed with 1% NP-40 with protease inhibitor cocktail (Roche) for 30 min. Lysates were incubated with anti-GFP antibody (Roche) at 4°C overnight. After incubation with protein G-Sepharose beads (Sigma) for 1 h, samples were washed twice with ice-cold 0.1% NP-40. Then beads containing YB-1-GFP were incubated with 10 µl reaction buffer at 37°C for 2 h and purified.

### Exonuclease or Exoribonuclease activity assay

The single-stranded oligonucleotides (21mer; Cosmo Genetech, Seoul, South Korea) and Poly-A tail RNA (12 mer; Bioneer, Seoul, South Korea) were end-labeled with [α-^32^P] ATP (Perkin Elmer, Waltham, MA) using T4 polynucleotide kinase (Thermo Scientific) at 37°C for 1 h and then heat inactivated at 75°C for 10 min. Purified YB-1 proteins were incubated with labeled oligonucleotides (30 pmol) or poly-A tail RNA (30 pmol) in a reaction mixture containing 10 mM Tris-HCl pH 8.0, 5 mM MgCl_2_, 50 mM KCl, 10 mM DTT, and 40 units of recombinant RNase inhibitor (TaKaRa) at 37°C for 2 h. The reactions were terminated by adding 2× loading buffer (90% formamide, 10 mM EDTA, 0.1% Xylene cyanol, 0.1% bromophenol blue) and boiling at 95°C for 1 min. After vortex and gentle centrifugation, samples were separated on a 15% polyacrylamide 7 M urea gel in TBE buffer at 70 V for 90 min and dried at 60°C for 1 h. Dried gels were exposed to film.

### TCA precipitation

Samples were precipitated with TCA(10% v/v), collected by centrifugation at 20,000 g for 45 min at 4°C, washed twice with ice-cold 70% ethanol, and then dried completely. Air-dried pellet were resuspended in distilled water.

### Flow cytometry

The surface expression of F4/80 or CD11c, which are lineage markers on macrophages or dendritic cells, was determined by flow cytometry (FACScalibur, Becton Dickinson Biosciences). Cells (1×10^6^) were washed twice with cold PBS containing 1% bovine serum albumin (BSA) and incubated for 1 h at 4°C with a saturating concentration of mAb F4/80 or CD11c (R&D Systems, Minneapolis, MN). Normal mouse IgG was used as a negative control for each test. The cells were washed twice with cold PBS containing 1% BSA and then stained with FITC-conjugated goat anti-mouse IgG for 50 min. A total of 10,000 gated events were collected by the FACScalibur cytometer and analyzed with CellQuest software (BD Biosciences).

### Real time PCR and RT-PCR assays

Total cellular RNA was prepared using an RNA prep kit (GeneAll, Seoul, South Korea) and RNA (0.5 µg) was reverse transcribed for 1 h with random hexamers at 42°C using M-MLV (Moloney Murine Leukemia Virus) reverse transcriptase (Enzynomic, Seoul, South Korea). PCR was then performed and PCR products were visualized on ethidium bromide-stained gels. Real time PCR was performed using TOPreal qPCR premix (SYBR Green, Enzymonics) and an Applied Biosystems 7300 Real-Time PCR System (Life technologies). Results were normalized to expression of the gene encoding GAPDH and were quantified by the change-in-threshold method (ΔΔCT). All primer sequences are listed in **Table S1**.

### Luciferase assay

Macrophages or dendritic cells were dispensed into each well of 24 well plates and were transfected with YB-1, renilla reporter gene, or IL-6 reporter plasmid. After 24 h, the transfected cells were stimulated by LPS for 12 h and lysed with luciferase buffer. The luciferase assays were performed with the Dual-luciferase Reporter Assay System (Pierce) and measured with a luminometer. All the luciferase assays were performed in triplicate for each luciferase reporter construct.

### Ethics statement

All animal experiments were performed in accordance with the Korean Food and Drug Administration (KFDA) guidelines. Protocols were reviewed and approved by the Institutional Animal Care and Use Committee (IACUC) of the Yonsei Laboratory Animal Research Center (YLARC). All mice were maintained in the specific pathogen-free facility of the YLARC.

## Supporting Information

Figure S1 **The surface expression of F4/80 or CD11c, which are lineage markers on macrophages or dendritic cells, was determined by flow cytometry.**
(TIF)

Table S1 **PCR primer sequences for RT-PCR or qRT-PCR.**
(DOCX)
