# Supplementary figures and images for: Differential Control of Interleukin-6 mRNA Levels by Cellular Distribution of YB-1
Source: PLoS One. 2014 Nov 14;9(11):e112754. doi: 10.1371/journal.pone.0112754 (PMC4232504; doi:10.1371/journal.pone.0112754)

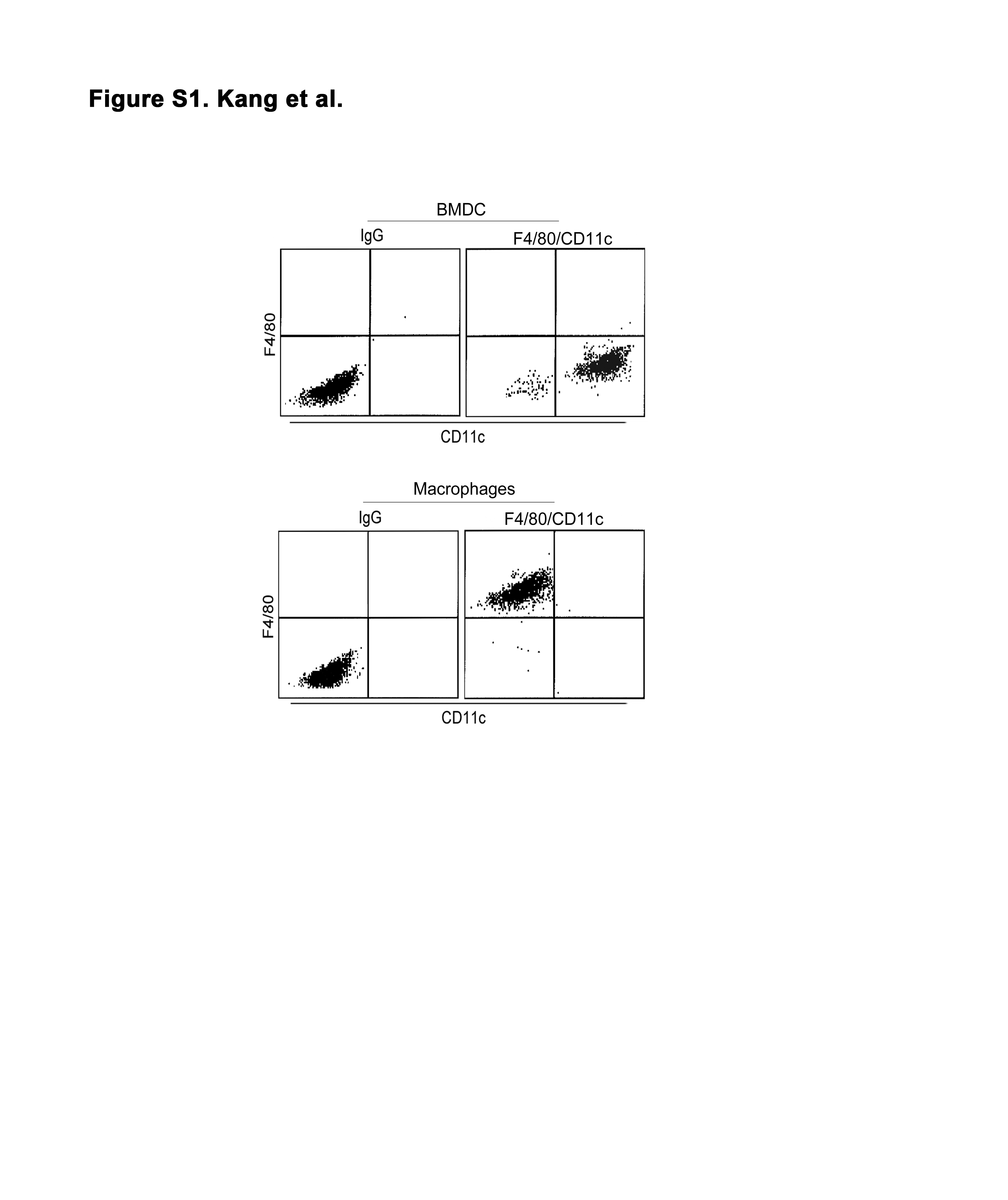

Supplement: Figure S1 — The surface expression of F4/80 or CD11c, which are lineage markers on macrophages or dendritic cells, was determined by flow cytometry. (TIF) [file pone.0112754.s001.tif]
