# Supplementary material for: Differential Control of Interleukin-6 mRNA Levels by Cellular Distribution of YB-1
Source: PLoS One. 2014 Nov 14;9(11):e112754. doi: 10.1371/journal.pone.0112754 (PMC4232504; doi:10.1371/journal.pone.0112754)
Supplement: Table S1 — PCR primer sequences for RT-PCR or qRT-PCR. (DOCX) [file pone.0112754.s002.docx]

**Table S1.** PCR primer sequences for RT-PCR or qRT-PCR

| Primer Number | Gene | Method | Forward Primer  Reverse Primer |
| --- | --- | --- | --- |
| P1 | IL-6 exon 5 | RT-PCR | 5′-GATTTACATAAAATAGTCCTTCCTACC-3′  5′-GGTTTGCCGAGTAGATCTCAAAGTG-3′ |
| P2 | IL-6 exon 4-5 | qRT-PCR | 5′- GTCCTTCAGAGAGATACAGAAACT-3′  5′- AGCTTATCTGTTAGGAGAGCATTG-3′ |
| P3 | YB-1 | RT-PCR  qRT-PCR | 5′-GGACAAGAAGGTCATCGCAACG-3′  5′-CCTACGACGTGGATAGCGTCTA-3′ |
| P4 | TNF-α exon 3-4 | RT-PCR | 5′-ACAAGCCTGTAGCCCACGTC-3′  5′-AAGACTCCTCCCAGGTATATGG-3′ |
| P5 | GAPDH | RT-PCR  qRT-PCR | 5′-CCTGGCCAAGGTCATCCATG-3′  5′-GCAGGAGACAACCTGGTCCT-3′ |
| P6 | 5’-UTR of IL-6 | RT-PCR | 5′-CCAAGAACGATAGTCAATTCCAGAAAC -3′  5′-ACCGGCAAGTGAGCAGATAGC -3′ |
| P7 | IL-6 exon1-2 | RT-PCR | 5′-ACCGCTATGAAGTTCCTCTCTGCA-3′  5′-AAGCCTCCGACTTGTGAAGTGGT-3′ |
| P8 | IL-6 exon2-5 | RT-PCR | 5′-CCACTTCACAAGTCGGAGGCT-3′  5′-AGCCACTCCTTCTGTGACTCC -3′ |
| P9 | 3’-UTR of IL-6 | RT-PCR | 5′-CCTAAGCATATCAGTTTGTGGACATTC-3′  5′-CTTATACATTCCAAGAAACCATCTGGC -3′ |
| P10 | HDAC6 mRNA | RT-PCR  qRT-PCR | 5′-ACCGGTATGACCGTGGCACT-3′  5′-TCCAGGGCACATTGACAGTGA -3′ |
